# Arcuate nucleus homeostatic systems reflect blood leptin concentration but not feeding behaviour during scheduled feeding on a high‐fat diet in mice

**DOI:** 10.1111/jne.12498

**Published:** 2017-08-25

**Authors:** T. Bake, J. Baron, J. S. Duncan, D. G. A. Morgan, J. G. Mercer

**Affiliations:** ^1^ Obesity and Food Choice Theme Rowett Institute University of Aberdeen Aberdeen UK; ^2^ AstraZeneca, Mereside Macclesfield UK; ^3^ Present address: Department of Physiology/Endocrinology Institute of Neuroscience and Physiology The Sahlgrenska Academy at the University of Gothenburg Gothenburg Sweden; ^4^ Present address: School of Pharmacy Keele University Staffordshire UK

**Keywords:** binge‐type eating, energy balance gene expression, mice, palatable diet, scheduled feeding

## Abstract

Hypothalamic homeostatic and forebrain reward‐related genes were examined in the context of scheduled meal feeding without caloric restriction in C57BL/6 mice. Mice fed ad libitum but allowed access to a palatable high‐fat (HF) diet for 2 hours a day rapidly adapted their feeding behaviour and consumed approximately 80% of their daily caloric intake during this 2‐hour scheduled feed. Gene expression levels were examined during either the first or second hour of scheduled feeding vs 24 hours ad libitum feeding on the same HF diet. Gene expression of neuropeptide Y, agouti‐related peptide, cocaine‐ and amphetamine‐regulated transcript, pro‐opiomelanocortin, long‐form leptin receptor and suppressor of cytokine signalling‐3 in the hypothalamic arcuate nucleus (ARC), as well as enkephalin, dynorphin, dopamine‐2‐receptor and dopamine‐3‐receptor in the nucleus accumbens (NAcc) in the forebrain, were measured by in situ hybridisation. Mice fed ad libitum on a HF diet had the highest total caloric intake, body weight gain, fat mass and serum leptin, whereas schedule‐fed mice had a mild obese phenotype with intermediate total caloric intake, body weight gain, fat mass and serum leptin. The effects of feeding regime on ARC gene expression were emphasised by significant positive or negative correlations with body weight gain, fat mass and blood leptin, although they did not appear to be related to feeding behaviour in the schedule‐fed groups (ie, the large, binge‐type meals) and did not reveal any potential candidates for the regulation of these meals. Mechanisms underlying large meal/binge‐type eating may be regulated by nonhomeostatic hedonic processes. However, assessment of opioid and dopamine receptor gene expression in the NAcc did not reveal evidence of involvement of these genes in regulating large meals. This complements our previous characterisation of ARC and NAcc genes in schedule‐fed mice and rats, although it still leaves open the fundamental question about the underlying mechanisms of meal feeding.

## 1 INTRODUCTION

Obesity has now assumed epidemic proportions, and arises when energy intake exceeds energy expenditure for a prolonged period, leading to storage of the excess energy as body fat.^1^, ^2^ Intense research efforts over the last two decades have established that the hypothalamus plays a key role in the regulation of energy homeostasis, where hypothalamic anorexigenic and orexigenic pathways are involved in the control of energy homeostasis in states of both chronic negative or positive energy homeostasis.^3^ Although energy balance itself is a homeostatic process, hypothalamic homeostatic systems might not play a preeminent role in the regulation of meal eating in obesity and may be over‐ridden by hedonic systems.^4^ Significantly, there is only weak evidence for a relationship between diurnal feeding patterns and diurnal expression profiles of hypothalamic genes regulating food intake.^5^ However, specific neuronal populations in hypothalamic areas involved in food intake regulation are activated in meal feeding schedules during the eating period itself but not in advance of it, suggesting that there is an active recruitment of these areas during the meal feeding process.^6^

Aiming to further determine the role of orexigenic and anorexigenic neuropeptide genes in driving meal feeding, we employed a palatable scheduled feeding model, as first described by Berner et al.,^7^ and based on dietary manipulations by Corwin et al.,^8^, ^9^ to induce a substantial food intake over short periods of time. Accordingly, providing scheduled access to a solid high‐fat (HF) palatable diet for a 2‐hour period each day, without imposed caloric restriction during the remainder of the day, resulted in consumption of large, binge‐type meals in both rats and mice.^10^ Despite the size of these feeding events and their relatively short duration, our previous examination of this model in the two species did not provide evidence of any potentially causative perturbation in the expression of hypothalamic homeostatic neuropeptide genes, in advance of the meal‐eating period, which might be driving the consumption.^10^ This finding was reinforced by the fact that the schedule‐fed rats and mice were not in a negative energy balance in advance of the initiation of the large meals (ie, did not express hypocaloric intake in the immediate run up to the scheduled feeding).^11^, ^12^ The aim of the present study was to follow up on these previous observations by further examining a potential role of hypothalamic homeostatic neuropeptide genes in driving meal feeding in mice, focusing on the scheduled feeding period itself, either the first hour of scheduled feeding or the second hour of scheduled feeding. Taking account of the established interactions between homeostatic centres in the hypothalamus and forebrain reward centres,^13^ expression levels of related genes in the nucleus accumbens (NAcc) were also assessed.

## 2 MATERIALS AND METHODS

### 2.1 Animals

Thirty‐two male C57BL/6 mice (Harlan, Bicester, UK) with initial body weights of approximately 23 g at 7 weeks of age were placed under a reversed 12 : 12 h light/dark cycle (lights on 15.00 hours, ZT0; lights off 03.00 hours, ZT12; ZT, zeitgeber time) immediately upon arrival and were individually housed after an acclimatisation period of 7 days in groups. All mice were fed ad libitum standard pellet diet [Special Diet Services, Witham, UK; #871505 CRM (P); 22% protein, 69% carbohydrate, 9% fat by energy, 2.67 kcal g^‐1^] unless otherwise noted. Water was freely available at all times during the experiments. All procedures were licensed under the Animals (Scientific Procedures) Act of 1986 and received approval from the Ethical Review Committee at the Rowett Institute.

### 2.2 Dietary manipulation

After an acclimatisation period of 14 days to the reversed light/dark cycle, mice were allocated to one of four weight matched groups of eight mice each (body weight 24.7±0.2 g). Dietary manipulations with a HF pellet diet (Research Diets, New Brunswick, NJ, USA, #D12492; 20% protein, 20% carbohydrates, 60% fat by energy, 5.24 kcal g^‐1^) were performed for 18 consecutive days: one group of mice had ad libitum access to HF diet for 24 hours a day (HF) and two groups had scheduled access to HF diet for 2 hours a day from ZT18 to ZT20 (6‐8 hours into the dark phase) and standard pellet diet in the remaining time (2h‐HF‐1st and 2h‐HF‐2nd). A control group had ad libitum access to standard pellet diet (CON). Food intake was measured manually every day in 2 and 22 hours bins for all groups, with the 2‐hour bin corresponding to the scheduled feeding period. On the final day of the study, intake was measured in 1‐ or 2‐hour bins depending on the experimental group. Therefore, mice were housed on grid floors and the food given and food left including spillage was weighed at the start and end of the scheduled feed for all groups. Body weight was measured three times a week. Mice were killed by decapitation under isoflurane anaesthesia on days 18 and 19 at intervals during the scheduled presentation of the HF diet. Termination time for schedule‐fed mice was either during the first hour of scheduled feeding from ZT18 to ZT19 (6‐7 hours into the dark phase; 2h‐HF‐1st) or during the second hour of scheduled feeding from ZT19 to ZT20 (7‐8 hours into the dark phase; 2h‐HF‐2nd). Consequently, four CON mice and four HF mice were terminated at each time interval. Because there was no effect of subgrouping by time interval (1st vs 2nd hour) for either CON or HF groups for any measure (*P*>.05 by *t*‐test), all data from the eight CON mice were pooled, as were data from the eight HF mice. Post mortem, a trunk blood sample was taken and processed to serum, body composition was measured using a magnetic resonance imaging scan system (Echo Medical Systems, Houston, TX, USA) and brains were dissected, frozen on dry ice and stored at −80°C until required.

### 2.3 Circulating hormones and metabolites

Serum samples were assayed in duplicate for leptin, insulin, glucose, triglycerides and non‐esterified fatty acid (NEFA). The hormone assays comprised commercially available kits. Leptin concentrations were measured using a mouse‐specific enzyme‐linked immunosorbent assay (ELISA) kit (#EZML‐82K; Millipore, Billerica, MA, USA). The sensitivity was 0.2 ng mL^‐1^ and the intra‐assay coefficient of variation (CV) was 3.13%. Insulin concentrations were also measured using a rat/mouse specific ELISA kit (#EZRMI‐13K; Millipore). The sensitivity of the assay was 0.2 ng mL^‐1^ and the intra‐assay CV was 2.25%. Glucose, triglycerides and NEFA were determined using the fully automated Konelab analyser (Thermo Fischer Scientific, Waltham, MA, USA). The sensitivities of the assays were 0.3, 0.05 and 0.01 mmol L^‐1^, respectively, with intra‐assay CVs of 0.87%, 1.62% and 1.06%, respectively.

### 2.4 Brain gene expression

Coronal brain sections through the hypothalamus and the forebrain were cut with a thickness of 15 μm at −20°C using a cryostat (Leica Biosystems, Milton Keynes, UK) and then collected onto sets of 10 slides for each animal. The sections were thaw‐mounted onto poly‐l‐lysine coated slides and stored at −80°C until analysis of mRNA expression. Sections from the hypothalamus included the arcuate nucleus (ARC) and spanned a region from approximately caudal −2.80 mm to rostral −1.70 mm relative to Bregma, and sections from the forebrain included the NAcc and spanned a region from approximately rostral 1.18 mm to caudal 0.26 mm relative to Bregma, according to the atlas of the mouse brain.^14^ Sections from each mouse were hybridised with antisense riboprobes generated from cloned DNA complementary to partial fragments of neuropeptide Y (NPY), agouti‐related peptide (AgRP), cocaine‐ and amphetamine‐regulated transcript (CART), pro‐opiomelanocortin (POMC), long‐form leptin receptor (OB‐Rb) and suppressor of cytokine signalling‐3 (SOCS3) for the ARC, and enkephalin (ENK), dynorphin (DYN), dopamine‐2‐receptor (D2‐R) and dopamine‐3‐receptor (D3‐R) for the NAcc.

Hypothalamic and forebrain mRNA expression was quantified by in situ hybridisation using established methods.^15^, ^16^, ^17^ Briefly, sections were first fixed using paraformaldehyde, acetylated and hybridised using ^35^S‐labelled antisense riboprobes in a formamide buffer at 58°C overnight. After hybridisation, sections were incubated in RNase A solution at 37°C, dehydrated and apposed to an autoradiographic film (BioMax MR; Kodak, Rochester, NY, USA) for 5‐28 days as appropriate. The mRNA levels were analysed and quantified from the autoradiographic images by densitometry using image proplus, version 7.0 software (Media Cybernetics, Bethesda, MA, USA). The integrated optical density of the hybridisation signal was measured using standard curves generated from ^14^C autoradiographic microscales (Amersham, Little Chalford, UK). Mean values for all mice were determined from four to six comparable sections spanning either the ARC or NAcc. Areas analysed within the NAcc were the combined accumbens core and shell.

### 2.5 Statistical analysis

Statistical analysis was performed with sigmaplot, version 12.0 (Systat Software, Chicago, IL, USA). Data were analysed by one‐way ANOVA to reveal overall effects of dietary manipulation or by Kruskal‐Wallis one‐way ANOVA on ranks when the data were not normally distributed and/or variances were not equal. Post‐hoc and planned comparisons were assessed with Student‐Newman‐Keul tests (SNK). Correlations between variables were analysed by Spearman rank order correlation. *P*<.05 was considered statistically significant. The data are presented as the mean±SEM.

## 3 RESULTS

### 3.1 Food intake

**Figure 1:**
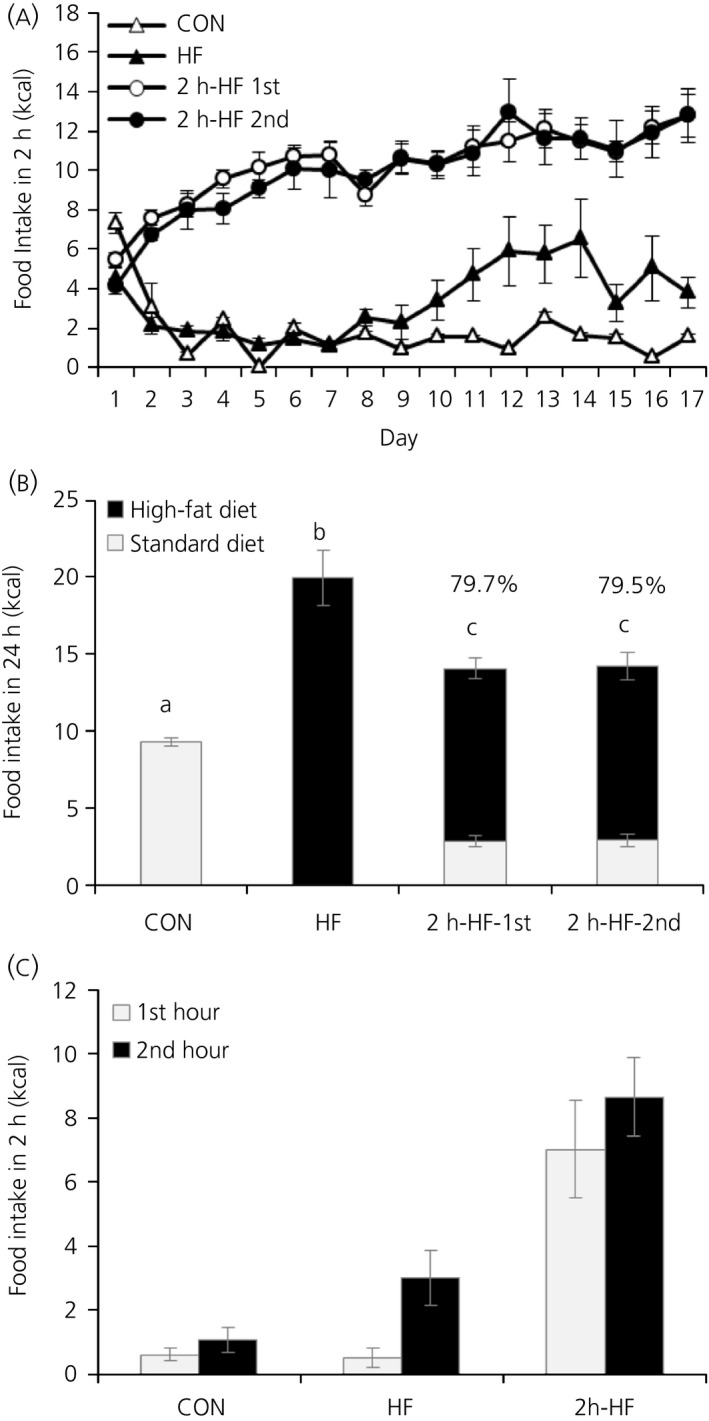
Caloric intake of C57BL/6 mice fed on a high‐fat diet with ad libitum or scheduled access. (A) Caloric intake during scheduled feeding time for all groups. (B) Total daily caloric intake during days 8 to 17 of the dietary manipulation showing calories consumed from standard diet and a high‐fat diet. Percentages above bars refer to calories consumed from high‐fat diet during scheduled feeding time relative to total 24‐hour intake. Pale grey bars, calories derived from control diet (CON); black bars, calories derived from a high‐fat (HF) diet. Different letters indicate *P*<.05 by one‐way ANOVA and Student‐Newman‐Keul post‐hoc test for total caloric intake. (C) Caloric intake during the scheduled feeding period on the day of sacrifice. Pale grey bars, intake during first hour of scheduled feeding; black bars, cumulative intake during the first and second hours of scheduled feeding. Data are presented as the mean±SEM

Both schedule‐fed groups quickly adapted their feeding behaviour to scheduled access conditions and showed large meal/bingeing behaviour (Figure 1A). In the second week of dietary manipulation, the calories consumed during scheduled feeding time were significantly higher in schedule‐fed mice than in CON and HF mice (SNK; *P*<.05); the percentages of total daily calories consumed as HF diet in the 2‐hour scheduled access period were 79.7% (11.2±0.7 kcal) and 79.5% (11.3±0.9 kcal) for 2h‐HF‐1st and 2h‐HF‐2nd schedule‐fed mice (Figure 1B). For comparison, CON mice consumed 15.6% (1.4±0.1 kcal) and HF mice consumed 20.1% (4.3±0.9 kcal) of total daily calories during the same 2‐hour period. During the 22 hours when the HF diet was not available, schedule‐fed mice significantly decreased their caloric intake on standard diet below that of CON mice (SNK; *P*<.05), although this compensation for HF diet caloric intake was incomplete, and total daily caloric intake was elevated in schedule‐fed mice (SNK; *P*<.05 vs CON), as well as in HF mice (SNK; *P*<.05 vs CON), compared to CON. On the final day of the study, most of the HF diet consumed during the scheduled feeding period was eaten during the first hour, and cumulative HF diet consumption at the end of the second hour was not significantly higher than at the end of the first hour (Figure 1C).

### 3.2 Body weight and composition

**Figure 2:**
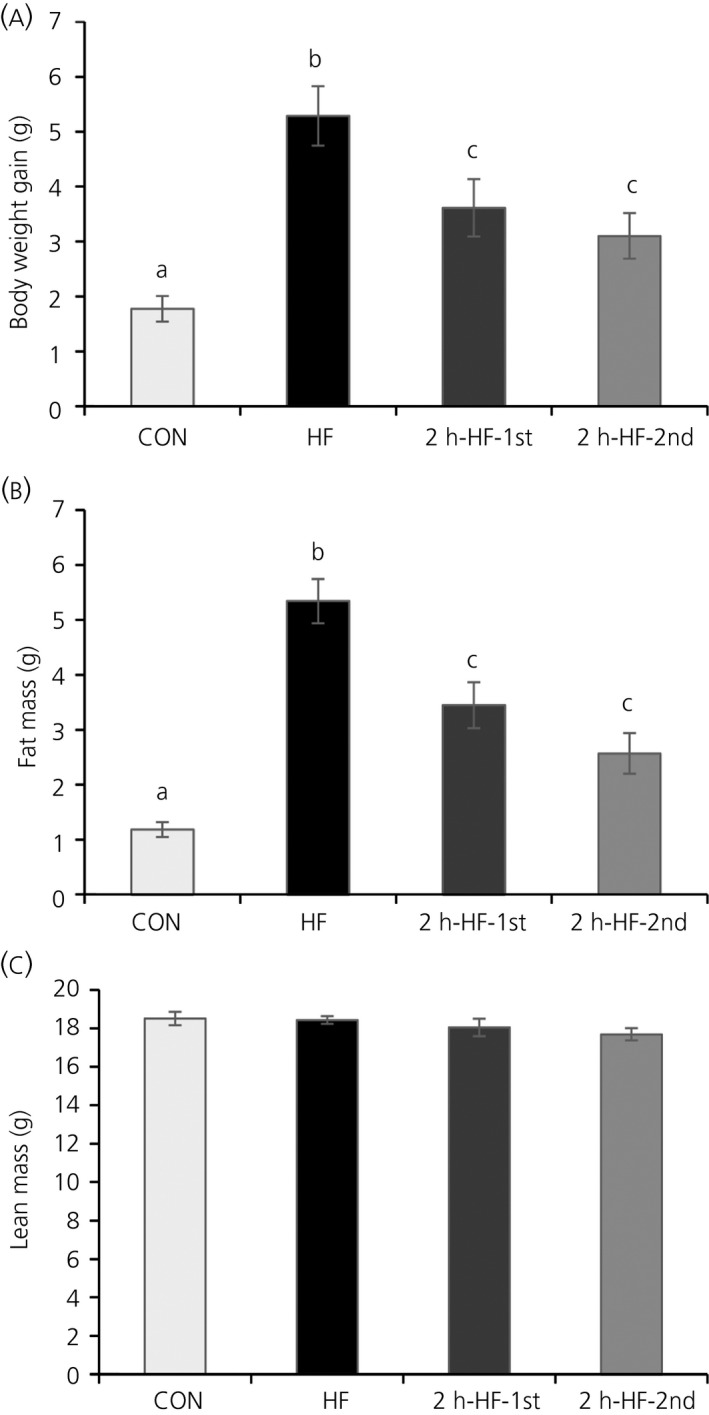
Body composition parameters of C57BL/6 mice fed on a high‐fat diet with ad libitum or scheduled access: (A) body weight gain (g), (B) body fat mass (g) and (C) body lean mass (g). Different letters indicate *P*<.05 by one‐way ANOVA and Student‐Newman‐Keul post‐hoc test. Data are presented as the mean±SEM. CON, standard pellet diet; HF, high‐fat

The dietary manipulation had significant effects on body weight gain and body fat mass (one‐way ANOVA; *P*<.001) (Figure 2A,B) but not body lean mass (Figure 2C). HF mice had the highest body weight gain (SNK; *P*<.001, *P*=.013 and *P*=.005 vs CON, 2h‐HF‐1st and 2h‐HF‐2nd) and body fat mass (SNK; *P*<.001 vs CON, 2h‐HF 1st and 2h‐HF 2nd), whereas both schedule‐fed groups had an intermediate body weight gain and body fat mass (SNK; *P*=.018 and *P*<.001 for 2h‐HF‐1st; *P*=.044 and *P*=.009 for 2h‐HF‐2nd vs CON).

### 3.3 Circulating hormones and metabolites

**Figure 3:**
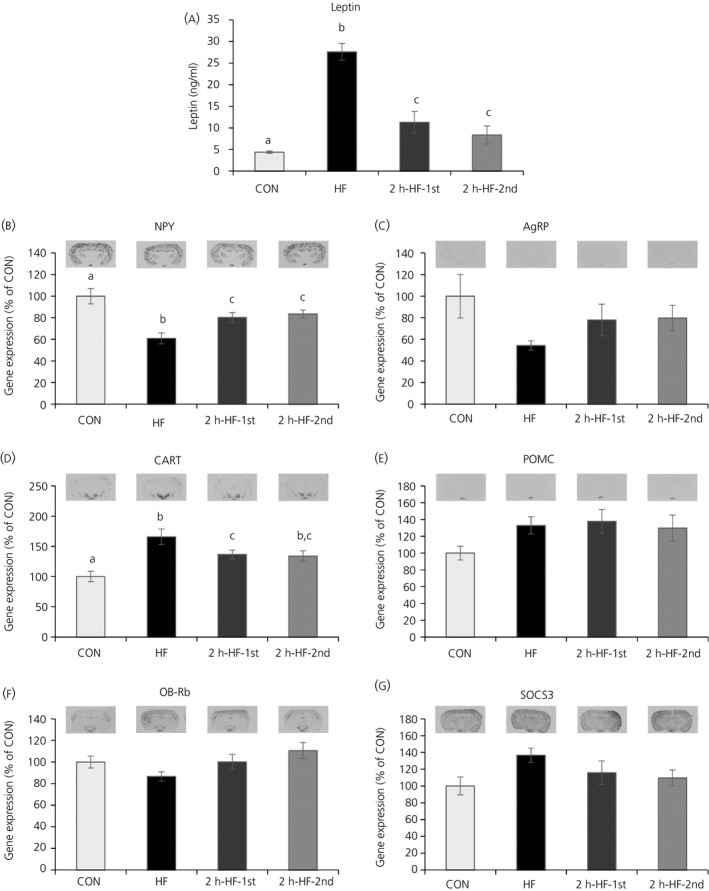
Quantification of mRNA gene expression in the arcuate nucleus of C57BL/6 mice fed on a high‐fat diet with ad libitum or scheduled access. (A) Serum leptin levels. (B‐G) Quantification of autoradiograph signals of (B) neuropeptide Y (NPY), (C) agouti‐related peptide (AgRP), (D) cocaine‐ and amphetamine‐regulated transcript (CART), (E) pro‐opiomelanocortin (POMC), (F) long‐form leptin receptor (OB‐Rb) and (G) suppressor of cytokine signalling‐3 (SOCS3), showing representative images used for quantification. Expression was normalised to the control group for each probe. Different letters indicate *P*<.05 by one‐way ANOVA and Student‐Newman‐Keul post‐hoc test. Data are presented as the mean±SEM. CON, standard pellet diet; HF, high‐fat

The hormones, leptin (Figure 3A) and insulin, as well as the metabolites, glucose, triglyceride and NEFA, were measured in the terminal blood sample (Table 1). One‐way ANOVA revealed significant effects of the feeding regime on leptin, glucose and NEFA concentrations (*P*<.001, *P*=.017 and *P*=.001, respectively). Leptin concentrations varied in parallel with total caloric intake, body weight gain and body fat mass, and were positively correlated with these parameters (*r*=.758, *r*=.784, *r*=.913, *P*<.001). HF mice had the highest leptin concentrations (SNK; *P*<.05 vs CON, 2h‐HF‐1st and 2h‐HF‐2nd), whereas intermediate concentrations were recorded in both schedule‐fed groups (SNK; *P*<.05 vs CON and HF). For insulin concentrations, there was a trend towards an effect of dietary manipulation (one‐ANOVA, *P*=.088), as well as a positive correlation with caloric intake during 2‐hour scheduled feeding time (*r*=.356, *P*=.045) and body weight gain (*r*=.434, *P*=.013). Glucose concentrations were lower in 2h‐HF‐2nd compared to CON (SNK; *P*=.023) and showed a trend to be lower compared to HF (SNK; *P*=.057); 2h‐HF‐1st showed a trend towards lower glucose concentrations compared to CON (SNK; *P*=.073). Glucose concentrations were negatively correlated with 2‐hour caloric intake during scheduled feeding time (*r*=−.443, *P*=.011) and positively correlated with 22‐hour caloric intake (*r*=.428, *P*=.015). NEFA concentrations were increased in HF and 2h‐HF‐2nd mice (SNK; *P*<.05 vs CON and 2h‐HF‐1st).

**Table 1:** Terminal blood hormones and metabolites after 18 days on dietary manipulation

	CON	HF	2h‐HF‐1st	2h‐HF‐2nd
Leptin (ng mL^‐1^)	4.38±0.27^a^	27.60±1.92^b^	11.28±2.49^c^	8.36±2.09^c^
Insulin (ng mL^‐1^)	2.46±0.34	2.95±0.25	4.17±0.73	3.83±0.56
Glucose (mmol L^‐1^)	10.02±0.42^a^	9.62±0.12^a,b^	8.78±0.52^a,b^	8.44±0.26^b,c^
Triglyceride (mmol L^‐1^)	1.16±0.12	1.04±0.09	0.79±0.10	1.16±0.16
NEFA (mmol L^‐1^)	0.68±0.04^a^	0.95±0.07^b^	0.59±0.05^a^	0.96±0.08^b^

Different letters indicate *P*<.05 by one‐way ANOVA and Student‐Newman‐Keul post‐hoc test. Data are presented as the mean±SEM. CON, standard pellet diet; HF, high‐fat; NEFA, non‐esterified fatty acid.

### 3.4 Energy balance and reward gene expression

For the energy balance neuropeptide genes, one‐way ANOVA revealed significant effects of the dietary manipulation on NPY and CART mRNA expression in the ARC (*P*<.001). HF mice had the lowest NPY mRNA expression (SNK; *P*<.001, *P*=.014 and *P*=.013 vs CON, 2h‐HF‐1st and 2h‐HF‐2nd) and both schedule‐fed groups had intermediate NPY mRNA expression (SNK; *P*=.032 and *P*=.034 vs CON) (Figure 3B). AgRP mRNA expression followed the same relative expression pattern as NPY mRNA expression, with a strong positive correlation (*r*=.534, *P*=.002) and with a trend towards an overall effect of dietary manipulation (one‐way ANOVA, *P*=.072) (Figure 3C). CART mRNA expression was highest in HF mice (SNK; *P*<.001, *P*=.037 and *P*=.060 vs CON, 2h‐HF‐1st and 2h‐HF‐2nd) and intermediate in both schedule‐fed groups (SNK; *P*=.028 and *P*=.017 vs CON) (Figure 3D). However, there were no significant effects on POMC mRNA expression (*P*=.152) (Figure 3E). For OB‐Rb mRNA expression, there was a trend towards an overall effect of the feeding regime (one‐way ANOVA, *P*=.074) (Figure 3F), as well as positive correlations with NPY and AgRP (*r*=.602, *P*<.001; *r*=.637, *P*<.001). There were no significant effects on SOCS3 mRNA expression (*P*=.131) (Figure 3G).

Serum leptin levels were negatively correlated with the orexigenic neuropeptide genes, NPY and AgRP, and also positively correlated with anorexigenic CART and SOCS3, and insulin concentrations were negatively correlated with NPY mRNA expression (Table 2). Furthermore, the neuropeptide genes showed strong correlations with total caloric intake, body weight gain and body fat mass (Table 2).

**Table 2:** Correlations of arcuate nucleus neuropeptide genes with blood hormones, caloric intake and body composition parameters

	NPY	AgRP	CART	POMC	OB‐Rb	SOCS3
Leptin	*r*=−.620, *P*<.001	*r*=−.366, *P*=.039	*r*=.613, *P*<.001	NS	NS	*r*=.488, *P*=.005
Insulin	*r*=−.442, *P*=.011	NS	NS	NS	NS	NS
Total caloric intake	*r*=−.488, *P*=.005	NS	*r*=.595, *P*<.001	*r*=.474, *P*=.006	NS	*r*=.598, *P*<.001
Body weight gain	*r*=−.686, *P*<.001	*r*=−.445, *P*<.011	*r*=.553, *P*=.002	NS	*r*=−.355, *P*=.046	NS
Body fat mass	*r*=−.674, *P*<.001	*r*=−.424, *P*=.016	*r*=.623, *P*<.001	NS	*r*=−.371, *P*=.037	*r*=.414, *P*=.019

Correlations between variables were analysed by Spearman rank order correlation. NPY, neuropeptide Y; AgRP, agouti‐related peptide; CART, cocaine‐ and amphetamine‐regulated transcript; POMC, pro‐opiomelanocortin; OB‐Rb, long‐form leptin receptor; SOCS3, suppressor of cytokine signalling‐3.

**Figure 4:**
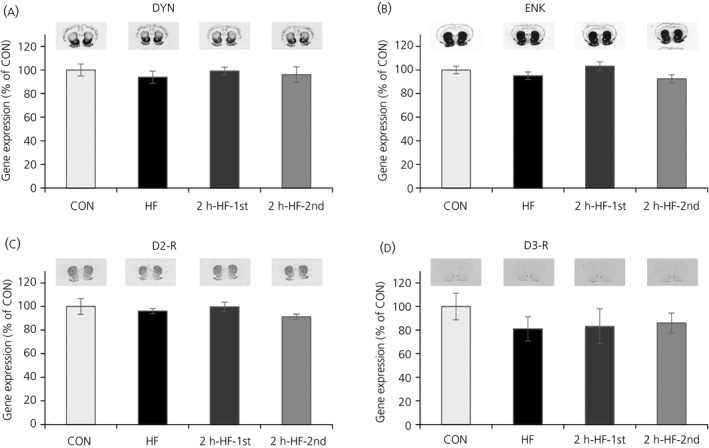
Quantification of mRNA gene expression in the nucleus accumbens of C57BL/6 mice fed on a high‐fat diet with ad libitum or scheduled access. (A‐D) Quantification of autoradiograph signals of endogenous opioids (A) dynorphin (DYN) and (B) enkephalin (ENK), and dopamine receptors (C) dopamine‐2‐receptor (D2‐R) and (D) dopamine‐3‐receptor (D3)‐R, showing representative images used for quantification. Expression was normalised to the control group for each probe. Data are presented as the mean±SEM. CON, standard pellet diet; HF, high‐fat

For the reward genes, there were no significant effects of the feeding regime on DYN, ENK, D2‐R or D3‐R mRNA expression in the NAcc (Figure 4).

## 4 DISCUSSION

The present study was designed to further assess the potential involvement of hypothalamic homeostatic systems and forebrain reward systems in driving the consumption of large palatable meals under a scheduled access paradigm vs ad libitum feeding. C57BL/6 mice fed ad libitum on a standard diet but allowed access to a palatable HF diet for 2 hours a day rapidly adapted their feeding behaviour and showed hyperphagia for the time that the palatable diet was available (Figure 1A), as already seen in our previous studies.^10^, ^12^ The resulting large binge‐type meals of HF diet were consistent with our previous work in rats^10^, ^11^ and in mice,^10^, ^12^ as well as with other published reports on this palatable scheduled feeding model in rats.^7^ Furthermore, this palatable feeding schedule promoted an intermediate increase in total caloric intake (Figure 1B) and a mild obese phenotype with moderately increased body weight gain (Figure 2A), body fat mass (Figure 2B) and blood leptin concentrations (Figure 3A). This feeding and body phenotype is also consistent with outcomes from our previous studies with the same HF diet in rats^10^, ^11^ and mice.^10^ Furthermore, the binge‐like eating behaviour seen in this animal model has relevance to the human situation. Binge‐eating disorder (BED) in humans, a clinical manifestation of binge‐eating,^18^ is strongly associated with obesity and is observed in up to 30% of patients seeking treatment for obesity.^19^, ^20^, ^21^, ^22^ It is obviously difficult to translate some aspects of human binge‐eating into a pre‐clinical model, especially subjective feelings such as emotional distress.^23^, ^24^ However, taking into consideration the objective criteria that are used to define BED,^23^ this schedule‐fed pre‐clinical model fulfils the following three criteria: (i) binge‐eating behaviour occurs repeatedly (ie, daily), over an extended period of time, as long as the dietary manipulation continues; (ii) binge‐eating animals consume more food in brief and discrete periods of time, in this case 2 hours, than relevant controls do under similar circumstances, that is, than either negative (CON) or positive (HF) control animals; and (iii) compensatory behaviour is initiated by the binge‐eating animal and not the investigator (ie, a voluntary decrease in standard diet is observed during the remainder of the 24‐hour period).^11^, ^12^ In addition, the model has relevance to eating in the absence of hunger, as is described for BED.^25^

In the present study, hypothalamic energy balance and forebrain reward‐related gene expression was examined during two intervals within the scheduled feeding period itself (2h‐HF‐1st and 2h‐HF‐2nd) and the responses to scheduled feeding were compared with those of mice fed ad libitum on the same palatable diet (HF). This modification in sampling time allowed assessment of potential correlates of the acute drive to binge on palatable HF diet during the scheduled feeding period itself as opposed to immediately prior to it, as previously investigated.^10^

Ad libitum HF mice in the present study were used as positive controls. This group had a phenotype typical of diet‐induced obesity (DIO), with increased total caloric intake (Figure 1B), body weight gain (Figure 2A), body fat mass (Figure 2B) and blood leptin concentrations (Figure 3A). HF mice also showed evidence of co‐ordinated regulation of homeostatic neuropeptide expression in the ARC, reflecting a state of positive energy balance, with a decrease in NPY, an increase in CART gene expression, and similar trends for AgRP and POMC, respectively (Figure 3). This regulation is typical of DIO, appears to be designed to counter further progression of obesity, and has previously been observed in mice on ad libitum HF diet feeding.^10^, ^26^, ^27^, ^28^

The homeostatic systems in the ARC have also been proposed to underpin the drive to eat under conditions of food restriction or fasting, and are considered to be the molecular basis of the phenomenon of hunger.^29^, ^30^ Mice under food restriction but prior to feeding, or mice under fasting conditions, show a pattern of ARC neuropeptide regulation the opposite of that of mice under DIO, with an up‐regulation of orexigenic neuropeptides, NPY and AgRP, and a down‐regulation of anorexigenic neuropeptides, CART and POMC.^31^, ^32^ Interestingly, even 2 hours after refeeding following long‐term restriction, mice still show the same pattern; however, 4 days ad libitum refeeding with hyperphagia abolishes the hunger signal and normalises gene expression levels.^31^ By contrast to the characteristic patterns underpinning the drive to eat under circumstances of negative energy balance, the acute drive to eat that is evidently present in schedule‐fed groups is accompanied by a gene expression profile more characteristic of ad libitum HF mice, with moderately decreased NPY and moderately increased CART gene expression, and appropriate trends in expression of other genes, suggestive of a longer‐term mechanism (Figure 3). This is in accordance with the results of our previous study**,** where it was not possible to show any perturbation of orexigenic and anorexigenic neuropeptides in the ARC in the 2‐hour period running up to scheduled feeding, and that could have been responsible for driving the large meals of HF diet.^10^ The value of investigating the homeostatic systems in the hypothalamus within the timeframe of the actual scheduled feeding period is underlined by studies using c‐fos to assess neuronal activation in the hypothalamus. Scheduled feeding studies employing a fasting/refeeding regime demonstrated that, during the feeding period, there was significant neuronal activation in rats^6^ and mice,^33^ as well as at the start of the feeding period in mice^34^; however, prior to the scheduled feeding period, there was only very little neuronal activation in rats.^6^ This suggests that there is an active involvement of hypothalamic neurones during the actual meal feeding process but not prior to a scheduled meal.

It appears that the homeostatic neuropeptide system in the ARC may not be directly involved in the regulation of large meals under nonrestricted conditions. Rather, homeostatic neuropeptide gene expression appears to reflect inappropriate body composition (ie, excess body adiposity), and perhaps excessive caloric intake, in both ad libitum HF and schedule‐fed mice. A relationship with the short‐term preceding feeding state in schedule‐fed mice appears less likely in the light of the numerous correlations between the gene expression levels and blood leptin concentrations, body weight gain, body fat mass and total caloric intake (Table 2). Furthermore, clear resolution of the involvement of the homeostatic system in the regulation of ad libitum feeding patterns on a standard diet is complicated by variability between studies. Although there is strong evidence that the network of anorexigenic and orexigenic neuropeptides in the hypothalamus is regulated by chronic negative^31^, ^32^ or positive^26^, ^27^, ^28^ energy balance in mice, the diurnal expression profiles of a panel of energy balance neuropeptides have been shown to have only a weak relationship with the ad libitum feeding pattern in a comprehensive study in C57BL/6 mice.^5^ Similar conclusions have been reached for some of the neuropeptides in ad libitum fed rats (eg, NPY^35^ and POMC^36^) , whereas AgRP in the latter study exhibited a diurnal rhythm broadly similar to the food intake pattern.^36^ However, in another study, NPY and POMC showed a daily rhythm in rats with the highest expression levels in the early‐ to mid‐light phase and the mid‐light phase, respectively.^37^ These inconsistencies serve to highlight the limits of our knowledge of the regulation of ad libitum feeding in general, as well as the role of energy balance genes in such circumstances, in particular. There are a number of notable scenarios where rather limited effects are observed on hypothalamic homeostatic neuropeptide expression: around the start of the dark phase in ad libitum fed animals when there is a sharp increase in food intake,^5^ prior to vs after refeeding in food restricted animals^31^ or in relation to food consumption vs no food consumption in food restricted animals.^38^ Taken together, this evidence suggests that hypothalamic homeostatic neuropeptides are not major players in at least the short‐term or more immediate regulation of food intake (ie, meal feeding).

An inference of the accumulating evidence that large, binge‐type meals are not being regulated by homeostatic neuropeptide systems in the ARC of the hypothalamus may be that these meals are regulated by hedonic processes.^10^, ^39^, ^40^ The amount of calories consumed voluntarily during the short time period certainly appears to be beyond homeostatic needs. Moreover, there are established interactions between the homeostatic centres in the hypothalamus and forebrain reward centres,^13^ and studies of other rodent models using restriction/refeeding cycles to induce binge‐like eating of palatable food have also suggested that at least binge‐eating itself is likely to be motivated by reward, rather than by metabolic need.^39^, ^40^ Previously, we were unable to demonstrate changes in gene expression levels of the endogenous opioid peptides, DYN and ENK, or the dopamine receptors, D2‐R and D3‐R, in the NAcc in the 2‐hour period running up to scheduled feeding in rats and mice.^10^ Because it is plausible that the hedonic systems might not be involved as early as 2 hours prior to a meal in a palatable feeding schedule, these NAcc genes were analysed during scheduled feeding on the palatable HF diet. However, no evidence of perturbations was found in DYN, ENK, D2‐R and D3‐R gene expression in the NAcc (Figure 4). There is evidence from other dietary manipulations to suggest that such regulation might be anticipated; NAcc genes might play a major role in mediating effects of rewarding food and also might be involved in the more immediate regulation of food intake. Studies employing restricted feeding schedules in mice have shown that there was a modest induction of neuronal activation in the NAcc.^34^ Rats with 3‐hour scheduled access to Ensure in addition to ad libitum chow exhibited a decrease in ENK mRNA 24 hours after the last Ensure exposure,^41^ and rats with a daily schedule of 12 hours of food deprivation and 12 hours of access to sucrose and chow showed a decrease in ENK and D2‐R mRNA and an increase in D3‐R mRNA.^42^ ENK in the medial accumbens shell, lateral accumbens shell and accumbens core has also been shown to be unaffected by chronic food deprivation, although it did track more immediate aspects of food consumption and short‐term satiety, with a down‐regulation by recent food consumption and an up‐regulation when food was withheld.^38^ It may be that analysis of expression of opioid precursor genes and dopamine receptors at the mRNA level is not an optimal approach with this precise dietary manipulation model, and that other approaches could be more productive in highlighting mechanistic underpinnings.

Between the first hour and the second hour of scheduled feeding, there were no differences in outcome at the level of gene expression. The only marked differences were in blood metabolites (Table 1). Mice terminated during the first hour (2h‐HF‐1st) had NEFA levels similar to CON, whereas mice terminated during the second hour (2h‐HF‐2nd) had elevated NEFA levels similar to HF. We have observed similar elevated NEFA levels in rats fed ad libitum on these HF diets,^10^ whereas schedule fed rats killed 1 hour before the scheduled HF access period had lower NEFA levels that were similar to CON rats. Similarly, rats fed ad libitum on a high energy diet^43^ had increased NEFA concentrations in the blood, most likely reflecting their higher body fat mass. Although high NEFA levels in ad libitum HF diet‐fed animals could reflect either dietary composition or body adiposity, NEFA in schedule‐fed mice show a time effect relative to meal start. The elevated NEFA levels in second‐hour schedule‐fed mice appear likely to be a time related absorption effect relative to meal start that is not present after 1 hour. Because schedule‐fed mice consume the majority of the HF diet during the first hour of schedule feeding (Figure 1C), the increase in NEFA during the second hour most likely reflects postprandial meal processing and arrival of fatty acids in the bloodstream. By contrast, blood glucose concentrations were lower in the 2h‐HF‐2nd group compared to CON, which was not the case for the 2h‐HF‐1st group, although a similar trend was observed. This observation may be comparable with the observed reduction in blood glucose across the scheduled feeding period in rats,^11^ and could reflect a metabolic adaptation for rapid tissue glucose uptake as the mice become habituated to bingeing on the HF diet, which is also relatively high in sucrose by energy.

## 5 CONCLUSIONS

In seeking to clarify the role of hypothalamic homeostatic systems in the ARC and forebrain reward systems in the NAcc in initiating meal feeding, we employed a scheduled feeding regime with 2 hours of daily access to a palatable HF diet in mice, based on the dietary manipulation described by Berner et al.^7^ Our data obtained during the scheduled feeding period itself in the present study, in conjunction with those obtained prior to the meal,^10^ suggest that the hypothalamic homeostatic systems do not have a substantive role in driving these palatable large, binge‐type meals, whereas counter‐regulatory changes were apparent in DIO mice with ad libitum access to the same palatable HF diet and, to a lesser extent, in the schedule‐fed groups. Thus, hypothalamic gene expression primarily reflects body composition in schedule‐fed mice, and reflects blood leptin concentration but not immediate feeding behaviour as assessed by 2‐hour caloric intake. The amount of calories consumed from the palatable HF diet in the 2‐hour access period was beyond immediate homeostatic needs, making it plausible that these events may be hedonically, rather than homeostatically, driven. However, it was not possible to demonstrate changes in NAcc gene expression for the candidate genes examined. Although the palatable scheduled feeding model is a valuable dietary manipulation because the mechanistic underpinnings of the behaviour exhibited are likely to be relevant to the over‐consumption of calories that underlies common human obesity, different experimental and analytical approaches may be required to reveal the underlying mechanisms.
